# 

**Published:** 1893-05-06

**Authors:** 


### CONTENTS.

**Recreation for Middle Age** - - - - - - - - - - 81

**Around the Hospitals** - - - - - - - - - - 82

**Medical History from the Earliest Times.** By E. T. WITHINGTON, M.A., M.B.Oxon. LIII.—Harvey and the New Physiology - - - - - - - - - - - - - 83

**Gleanings in Science and Medicine** - - - - - - 84

**The Drink Question in Switzerland** - - - - - - 85

**Contemporary Theory and Research** - - - - - - 86

**Annotations.**—The "Reckoning"—Leeds and Her Back-to-Back Houses—A "Cure" for Snoring (?) - - - - - - - 87

**The Hospital Clinic**—ROYAL SOUTHERN HOSPITAL, LIVERPOOL.—Treatment of Tubercular Joints - - - - - - - 89

ROYAL INFIRMARY, EDINBURGH.—The Treatment of Injuries to the Scalp - - - - - - - - - - - - 90

HUDDERSFIELD INFIRMARY.—Senile Gangrene - - - - 91

**Notes and News** - - - - - - - - - - - - 88

**Books Received** - - - - - - - - - - - - 92

**The Institutional Workshop**—

WITHIN THE HOSPITALS.—The North-Eastern Hospital for Children - - - - - - - - - - - - - 93

THE STORY OF THE INSANE FROM YEAR TO YEAR.—Cheshire County Asylum at Macclesfield - - - - - - - 94

HOSPITAL CONSTRUCTION.—Leith Fever Hospital - - - 94

FESTIVAL DINNERS.—The Metropolitan Hospital; The Richmond Hospital - - - - - - - - - - - - - 95

NEW DRUGS, APPLIANCES, AND THINGS MEDICAL.—New Sponge-Holding Electrode - - - - - - - - - - - 95

CONSTRUCTION NOTES - - - - - - - - - - - 96

**Scraps and Gleanings** - - - - - - - - - - 96

**The Book World of Medicine and Science** - - - - vii

### EXTRA SUPPLEMENT.—THE NURSING MIRROR.

**News from the Nursing World.**—A Royal Visit—Provision for Old Age—The Nurse Dolls—A Welcome Gift—Unattainable Training—Queen's Nurses at Portsmouth—Well-earned Honours—Self Help—Old and Incompetent—Training in Fever Nursing—District Nursing at Melbourne - - li

**Sanitation for Nurses.** By A SANITARY INSPECTOR. IV.—Sweden- - - - - - - - - - - - - - liii

**A German Hospital.** By AN ENGLISH PATIENT. II.—Doctor and Dinner - - - - - - - - - - - liv

**District Nurses** - - - - - - - - - - - - lv

**St. Mary's, Plaistow.**—Opening of the New Day Nursery - lvi

**Wants and Workers** - - - - - - - - - - - lvi

**Everybody's Opinion.**—Nurse Snorers and Others—The Lifting of Patients - - - - - - - - - - - lvi

**Quarantine for Nurses** - - - - - - - - - - lvii

**Appointments—Where to Go** - - - - - - - - lvii

**For Reading to the Sick.**—Prescription. III. - - - - lvii

**Her Better Self.** III.—"Far Above Rubies" - - - - lviii

**Notes and Queries** - - - - - - - - - - - lviii

**The Book World for Women and Nurses** - - - - lix

